# Environmental and Production Aspects of Using Fertilizers Based on Waste Elemental Sulfur and Organic Materials

**DOI:** 10.3390/ma15093387

**Published:** 2022-05-09

**Authors:** Aneta Lisowska, Barbara Filipek-Mazur, Monika Komorowska, Marcin Niemiec, Dominika Bar-Michalczyk, Maciej Kuboń, Sylwester Tabor, Zofia Gródek-Szostak, Anna Szeląg-Sikora, Jakub Sikora, Sławomir Kocira, Zbigniew Wasąg

**Affiliations:** 1 Institute of Technology and Life Sciences, National Research Institute, Falenty, 3 Hrabska Av., 05-090 Raszyn, Poland; a.lisowska@itp.edu.pl (A.L.); d.michalczyk@itp.edu.pl (D.B.-M.); 2 Department of Agricultural and Environmental Chemistry, University of Agriculture in Krakow, 21 Mickiewicza Av., 31-120 Krakow, Poland; barbara.filipek-mazur@urk.edu.pl (B.F.-M.); monika.komorowska@urk.edu.pl (M.K.); 3 Faculty of Production and Power Engineering, University of Agriculture in Krakow, 30-149 Krakow, Poland; maciej.kubon@urk.edu.pl (M.K.); anna.szelag-sikora@urk.edu.pl (A.S.-S.); jakub.sikora@urk.edu.pl (J.S.); 4 Department of Production Engineering, Logistics and Applied Computer Science, Faculty of Production and Power Engineering, University of Agriculture in Krakow, Mickiewicza 21, 30-120 Krakow, Poland; sylwester.tabor@urk.edu.pl; 5 Department of Economics and Enterprise Organization, Cracow University of Economics, 31-510 Krakow, Poland; grodekz@uek.krakow.pl; 6 Institute of Management and Production Engineering, Cavalry Captain Witold Pilecki State University of Małopolska in Oświęcim, Maksymiliana Kolbego 8, 32-600 Oswiecim, Poland; 7 Department of Machinery Exploitation and Management of Production Processes, University of Life Sciences in Lublin, Akademicka 13, 20-950 Lublin, Poland; slawomir.kocira@up.lublin.pl; 8 Jan Zamoyski College of Humanities and Economics in Zamość, ul. Koszary 8, 22-400 Zamość, Poland; zbigniew.wasag1@wp.pl

**Keywords:** waste sulfur, sulfate sulfur, pH, organic matter, soil enzymatic activity, management, sustainability development

## Abstract

Crop fertilization with sulfur is an important part of agricultural practices, as is the systematic increase in soil organic matter content. Materials of waste origin constitute a source of plant-available sulfur, as well as soil organic matter. The study was to verify the hypothesis assuming that combining waste sulfur pulp and its mixtures with organic materials enables simultaneous soil enrichment with readily available sulfur and organic matter. A 240-day incubation experiment was conducted, on two soils: very light and heavy; with two sulfur doses applied to each soil (20 and 40 mg S/kg d.m. for very light soil, and 30 and 60 mg S/kg d.m. for heavy soil). The sulfate sulfur content in the incubated soil material, treated with the addition of sulfur pulp and its mixtures with organic materials, increased significantly up to day 60 and then decreased. The application of these materials significantly increased the content of available sulfur and decreased the pH value of the incubated material. The effect of the introduced materials on dehydrogenase activity depended on soil granulometric composition (the impact of the applied materials on the activity of these enzymes in very light soil was small, and in heavy soil, their activity was usually limited by the presence of introduced materials). Application of the studied materials had little effect on the total organic carbon content in the incubated soil material (a significant change in the value of this parameter, in relation to the control soil, was recorded in some treatments of heavy soil).

## 1. Introduction

Sulfur is the 14th most commonly occurring element on Earth [1]. It occurs in mineral and organic formations in rocks, soil, water, the atmosphere, and the biosphere. Plants take up sulfur from the soil solution, mainly as sulfate ion SO_4_^2−^. This nutrient plays a significant role in many processes taking place in plant cells, including: participation in redox reactions, detoxification of heavy metals, synthesis of carbohydrates and lipids, formation of chlorophyll, and photosynthesis process, supporting plants’ responses to biotic and abiotic stresses [2,3,4,5,6,7,8].

In the middle of the 19th century, natural sulfur circulation was disturbed by human activity. The development of industry, as well as intensive coal combustion, increased the emission of sulfur oxides into the atmosphere, and finally environmental pollution [1,9,10]. Due to this, the amount of sulfur emissions started to be controlled. Conducted for many years, pro-ecological activities resulted in a significant reduction in the amount of this element’s compound deposition. Nowadays, sulfur deficiency in the soil environment has been observed, which results (among other things) from the practices mentioned above. A disturbance of sulfur balance in the environment affects many countries in Europe, North America, China, and India, where the average sulfur share is insufficient to cover the nutritional requirements of most plant species. According to the estimates, this situation will last at least until 2050 [11,12,13].

There are several ways to supplement soil sulfur stock: mineral, organic, and natural fertilizers, green manures, crop residues, and waste materials. Agricultural reuse of waste materials can bring environmental benefits (a decrease in the use of conventional mineral fertilizers, production of which requires input of non-renewable resources, a decrease in the amount of waste deposited in landfills) and is consistent with the idea of a circular economy. This approach aims at sustainable resource management and a reduction in the environmental burden of human activity [14,15,16,17,18,19].

Another significant issue is the low or decreasing content of soil organic matter. This extremely important soil component shapes its physical, chemical, and biological properties. In addition, soil organic matter constitutes a key element of the global carbon cycle as its significant reservoir. Some 45% of European soils are characterized by low and very low content of organic matter (0–2% organic carbon). Such soils are threatened with desertification, which is why it is recommended to increase organic matter resources in such habitats. Maintaining a positive balance of this component is essential to preserving productional and environmental soil functions [20,21].

Environmental degradation and climate change, caused by human activity, is one of the most dangerous issues the present world is facing [22,23,24]. Due to the rate at which these changes take place, living organisms, as well as whole natural habitats, are not able to adapt to the new conditions. Increasing erosion, loss of organic carbon resources, an imbalance of nutrients, salinity, land conversion, loss of biodiversity, pollution, acidification, compaction, floods, and droughts are the main factors threatening soil fertility. Therefore, it is necessary to verify the used soil management practices, since soil is a non-renewable resource and the main component of natural habitats [21]. The increase in organic matter and sulfur stocks in soil is an important issue, not only on a local but also on a global scale. Furthermore, waste elemental sulfur could be an alternative supplementary source of the element. This method of sulfur waste reuse corresponds with the upcoming trend to create zero-waste technologies in all economies [25,26,27]. For this reason, model research was conducted to verify the hypothesis, assuming that the application of waste sulfur pulp and its mixtures with organic materials enables simultaneous soil enrichment with readily available sulfur and organic matter.

## 2. Materials and Methods

### 2.1. Properties of the Soil Material

The soil material was collected in southern Poland. The study included two soils sourced from a 0–20 cm layer: the first one was very light (sand), and the second one was heavy (silt loam). To prepare the soil material for the experiment, it was air-dried and sifted. Before setting up the experiment, very light and heavy soil had acid and very acid reactions, respectively. Both soils (very light and heavy) had low sulfate sulfur and total sulfur content and were free from heavy metal contamination (Table 1).

Four weeks prior to setting up the incubation experiment, liming treatment was conducted. CaCO_3_ (Fluka, Buchs, Switzerland) and CaO (POCH, Gliwice, Poland) were applied to very light and heavy soil, respectively (at a dose corresponding to 0.75 of hydrolytic acidity—for very light soil: 313 mg CaO/kg d.m. of soil; for heavy soil: 873 mg CaO/kg d.m. of soil). After that, soil material moisture was maintained at 60% of the maximum water capacity level and a temperature of 25 ± 2 °C. As a result of conducted treatment, very light and heavy soil was characterized by a slightly acid (pH_H2O_ = 6.55, pH_KCl_ = 5.78) and acid reaction (pH_H2O_ = 5.69, pH_KCl_ = 4.95), respectively.

### 2.2. Model Incubation Experiment

The incubation experiment design included two soils. For each soil, there were nine treatments (each treatment was conducted in three replications):

1. Control soil (without additions)—C;
2. Soil with the addition of sulfur pulp (sulfur dose: I)—SI;
3. Soil with the addition of sulfur pulp (sulfur dose: I) and manure—SI + M;
4. Soil with the addition of sulfur pulp (sulfur dose: I) and digestate—SI + D;
5. Soil with the addition of sulfur pulp (sulfur dose: I) and biochar—SI + B;
6. Soil with the addition of sulfur pulp (sulfur dose: II)—SII;
7. Soil with the addition of sulfur pulp (sulfur dose: II) and manure—SII + M;
8. Soil with the addition of sulfur pulp (sulfur dose: II) and digestate—SII + D;
9. Soil with the addition of sulfur pulp (sulfur dose: II) and biochar—SII + B.

Two sulfur doses (SI and SII) were considered for each soil (for very light soil: 20 (SI) and 40 mg S/kg d.m. of soil (SII); for heavy soil: 30 (SI) and 60 mg S/kg d.m. of soil (SII)). To determine the sulfur doses, the sulfate sulfur content in the soil material prior to establishing the incubation, as well as the Polish guidelines regarding the assessment of sulfur content in soils, were included. Soil without any additions was regarded as control (C). In addition to sulfur pulp, organic materials (manure (M), digestate (D), and biochar (B)), were used. The doses of these materials were determined according to the assumption to apply the same dose of total carbon to each of them. To very light and heavy soil, 1000 and 1500 kg C per 1 ha was introduced, respectively. These doses would correspond to about 100 and 150 kg of introduced nitrogen, respectively. All of the used materials, sulfur pulp, manure, digestate, and biochar, were dried and ground to be mixed properly with soil particles.

**Figure 1:**
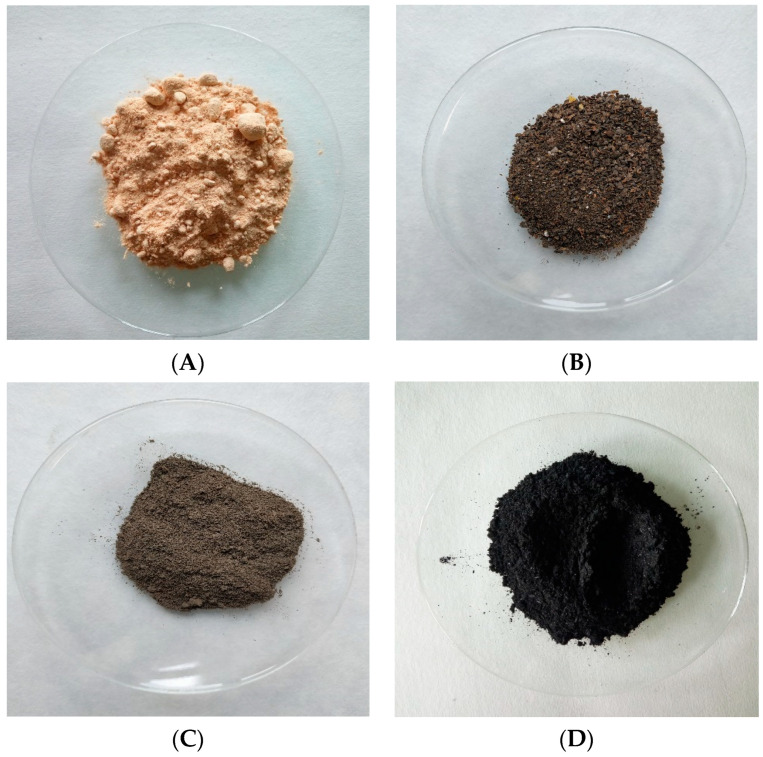
Materials used in the incubation experiment: (**A**) sulfur pulp, (**B**) manure, (**C**) digestate, (**D**) biochar.

As a source of sulfur, the waste sulfur pulp (Figure 1A) was used. This material was produced in a sewage sludge treatment plant during biogas purification (obtained from sewage sludge methane fermentation) via Biosulfex^®^ technology (PROMIS Company, Warsaw, Poland), involving iron and EDTA ligand. The sulfur content (in elemental form S^0^) in this material exceeded 80% d.m. (Table 2). As a source of organic matter, granulated cattle manure (Figure 1B), digestate (Figure 1C), and biochar (Figure 1D) were used. The manure used in the experiment was produced from fermented cattle manure, which was dried and then pressed into a pellet.

Digestate constituted a by-product of raw municipal sewage sludge anaerobic methane fermentation. Biochar was created as a result of anaerobic carbonization of plant biomass and was characterized by a high content of total carbon of 516 g/kg d.m. (Table 2). The waste materials (sulfur pulp, digestate) used in the conducted experiment came from a facility located in central Poland, while manure and biochar constituted commercially available products.

Soils were incubated in plastic containers. Each container included 280 g d.m. of very light soil or 200 g d.m. of heavy soil enriched with selected materials, according to the experimental design. Throughout the experimental period, soil moisture and temperature were maintained at 60% of the maximum water capacity level and 25 ± 2 °C, respectively. Soil samples for laboratory analyses were collected on the day the materials were applied as well as 15, 30, 60, 120 and 240 days after application. Collected soil samples were dried and sieved (1 mm mesh) to prepare for laboratory analyses. Laboratory analyses included the determination of pH value, sulfate sulfur, and total organic carbon content.

### 2.3. Methods of Laboratory Analyses

Soil pH_KCl_ was determined potentiometrically in a 1 mol/L potassium chloride (Chempur, Piekary Śląskie, Poland) suspension (1:2.5 *m*/*v*), using the CPC-502 (Elmetron, Zabrze, Poland) multifunction device. To calculate the value of mean pH, the pH values of three replicates were converted into hydrogen ion [H^+^] concentrations. Next, the arithmetic mean was calculated and converted into pH according to the equation: pH = −log[H^+^]. Sulfate sulfur (S-SO_4_) was extracted from the soil samples using a 0.03 mol/L acetic acid solution (Chempur, Piekary Śląskie, Poland) (30 min, 40 rpm, *m*/*v* 1:10). The elemental composition of the obtained extracts was determined using an Optima 7300 DV (Perkin-Elmer, Waltham, MA, USA) inductively coupled plasma optical emission spectrophotometer (ICP-OES method). The total organic carbon (TC) content was determined by the Turin method, based on the oxidation of organic compounds in acidic conditions. The dehydrogenase (DEH) activity was determined by transforming colorless, water-soluble 2,3,5-triphenyltetrazolium chloride (TTC) into red water-insoluble 1,3,5-triphenylformazan (TPF) [28]. The soil material was incubated with a 1.0% TTC (EUROCHEM BGD, Tarnow, Poland). TTC was prepared in a tris(hydroxymethyl) aminomethane hydrochloride (TRIS-HCL) buffer, pH 7.4. After incubation of the prepared samples (1:1 *m*/*v*, 96 h, 30 °C), TPF was extracted with 20 mL of methanol (Chempur, Piekary Slaskie, Poland) and quantified by the colorimetric method at the wavelength of 485 nm on a UV/VIS DU 640 spectrophotometer (Beckman, Fullerton, CA, USA).

To characterize the properties of the soils and introduced materials (sulfur pulp, manure, digestate, biochar) before the experiment, additional analyses were conducted. Soil granulometric composition was determined by the Bouyoucos–Casagrande’s areometric method in Proszynski’s modification [29]. The maximum water capacity of the soils was determined by measuring the difference in soil mass before and after moisture conditioning by capillary rise. The soil pH_H2O_ was determined in a water suspension (*m*/*v* 1:2.5) using potentiometric titration. Hydrolytic acidity was assessed by the Kappen method after extraction with 1 mol/L sodium acetate solution (1 h, 40 rpm, 2:5 *m*/*v*). The total content of carbon and nitrogen in all materials (soils, sulfur pulp, manure, digestate, and biochar) was determined using a Vario MAX cube CNS analyzer (Elementar Analysensysteme GmbH, Langenselbold, Germany). The total sulfur content in the soil material was determined after binding sulfur by magnesium nitrate (Merck KGaA, Darmstadt, Germany), dry mineralization (12 h, 450 °C), and dissolving the residue in a nitric acid solution (POCH, Gliwice, Poland). The total sulfur content in the manure, digestate, and biochar was determined after material oxidation by concentrated nitric acid (POCH, Gliwice, Poland), sulfur binding by magnesium nitrate (Merck KGaA, Darmstadt, Germany), dry mineralization (2 h, 300 °C then 3 h, 450 °C) and dissolving the residue in a nitric acid solution (POCH, Gliwice, Poland). The total content of other macroelements (Ca, P, Na, K, Mg) and trace elements (Fe, Ni, Pb, Zn, Cu, Cd, Mn, Cr) were determined after material incineration (8 h, 450 °C), evaporation with a mixture of concentrated acids: nitric acid (POCH, Gliwice, Poland) and perchloric acid (EUROCHEM BGD, Tarnów, Poland), and dissolving the residue in hydrochloric acid (Chempur, Piekary Śląskie, Poland). The total content of sulfur, other macroelements, and trace elements in the sulfur pulp was determined after digestion in a mixture of concentrated acids: hydrochloric acid (Chempur, Piekary Śląskie, Poland) and nitric acid (POCH, Gliwice, Poland) (3:1 *v*/*v*) (PN-EN 16964: 2018-03). The content of the analyzed elements in the solutions was determined by ICP-OES. The total mercury content in the materials (excluding sulfur pulp) was determined on an AMA-254 (Altec Ltd., Praha, Czech Republic) apparatus. Dry matter of sulfur pulp, manure, digestate, and biochar was determined using the weight method, from the difference in weight of the sample before and after drying. The organic matter content in manure, digestate, and biochar was determined by measuring the amount of ignition loss (4 h, 500 °C).

### 2.4. Statistical Analysis

The results were statistically analyzed to obtain the arithmetic mean and standard deviation (SD). We performed a one-way analysis of variance for a repeated measures system (qualitative factor: object; repeated measures factor: days of incubation; the number of repeated measures factor levels: 6), with the use of Dell Statistica, version 13 software for data analysis (Dell Inc., Tulsa, OH, USA). The significance of differences in mean values was assessed using Duncan’s test, with a significance level of *p* ≤ 0.05.

## 3. Results and Discussion

### 3.1. Value of Soil pH_KCl_

The functionality of the soil ecosystem is impacted by biological, chemical, and geological processes occurring in the pedosphere. Soil pH constitutes the main variable regulating the direction and rate of processes occurring in the pedosphere [30]. This parameter describes a degree of soil acidification or alkalization and represents hydrogen ions (H^+^) concentration in soil solution [31]. The scale of pH values is represented by a logarithmic scale, and a decrease of 0.60 units of pH value corresponds to a fourfold increase in hydrogen ions activity [32]. On the day of sulfur pulp and organic materials application, the pH_KCl_ value of very light soil ranged from 5.76 to 6.09, and heavy soil ranged from 4.95 to 5.08 (Table 3). During incubation, the pH value of both soils decreased. After 240 days of the experiment, the pH_KCl_ value of very light and heavy soil ranged from 5.02 to 5.31 and from 4.42 to 4.56 (Table 3), respectively.

After the conducted experiment, the effect of the sulfur dose on the pH_KCl_ value of both tested soils (regardless of the organic material addition) was observed. Throughout the incubation period, pH values of treatments with the addition of sulfur pulp at dose SII and its mixtures with organic materials were comparable to or significantly lower than the pH values of treatments with the addition of sulfur pulp at dose SI and its mixtures with organic materials.

As for the treatments fertilized only with sulfur pulp, it was found that organic materials had a beneficial effect on the pH value of both tested soils. As a rule, after the introduction of organic materials, comparable or significantly higher values of this parameter were determined. The increase in soil pH concerned mainly treatments with the addition of manure. After the introduction of digestate and biochar, in some samples, comparable or significantly lower values of soil pH were observed, than after the application of sulfur pulp alone.

The findings presented in this study indicate that the application of sulfur pulp and its mixtures with organic materials affected the value of pH_KCl_ of both tested soils. The decrease in soil pH after the addition of elemental sulfur results from the increasing concentration of hydrogen ions (H^+^), formed during its microbiological oxidation. The level of soil acidification depends on the dose of applied elemental sulfur and soil buffering capacity. Karimizarchi et al. [33] found that the degree of soil acidification increases with the increase in the S^0^ dose. Meanwhile, Yang et al. [34] presented that the application of 0.15 g S^0^/10 g d.m. of soil resulted in a decrease of soil pH by nearly 4 units, after conducting an 84-day incubation experiment. Tabak et al. [35], after the introduction of 10, 20, 30, and 60 mg S^0^/kg d.m. of soil, found that this treatment had slightly affected the soil pH (during a 120-day incubation experiment). Similar results were presented by Lisowska et al. [36], after the introduction of 20 mg S^0^/kg d.m. of soil and soil incubation for 90 days. Zhou et al. [37] found that the application of 2.00 mg S^0^/kg did not affect the incubated soil’s pH. The authors explained that this could be due to a high soil buffering capacity and low oxidation level of the introduced elemental sulfur. A decrease in soil pH, after S^0^ application, was also observed by Mattiello et al. [38] and Yang et al. [39]. The authors also stated that liming treatment reduces the negative impact of S^0^ on soil pH, and is an effective practice for maintaining this parameter at an optimal level.

In the conducted research, no clear positive impact of the introduced organic materials on the pH of the incubated soil (excluding heavy soil with the addition of sulfur pulp at sulfur dose I and manure treatment) was observed, compared to the control treatment. However, as a rule, applied organic materials reduced the acidifying effect of added elemental sulfur. Various findings, focusing on the effects of organic materials on soil pH, have been reported. Barłóg et al. [40], after conducting a 4-year field experiment, found that the application of digestate (at a dose of 20 Mg/ha/year) had no effect on the soil pH. Opposite results were presented by Tambone and Adani [41]. The authors, after conducting an incubation experiment, found that soil introduction of digestate, stabilized sewage sludge, compost (from a mixture of lignocellulosic residues and organic solid waste fraction), and mineral fertilizer (at a dose corresponding to 300 kg N/ha), resulted in a decrease in soil pH of all treatments. By contrast, Cai et al. [42] found that fertilization with manure prevented or reversed soil acidification after 18 years of using mineral fertilizers. Similar results were presented by L’Herroux et al. [43] and Whalen et al. [44]. An increase in soil pH, after manure introduction, may result from the presence of Ca^2+^ and Mg^2+^ basic cations and organic acids, which are able to neutralize H^+^ ions [43], according to Whalen et al. [44]. Studies have also shown negative effects of manure on soil pH [45], resulting from organic matter decomposition and the release of humic and fulvic acids, decreasing the value of this parameter [45,46]. Hailegnaw et al. [47], after amending soil with biochar at a dose of 0.5%, reported that this treatment had no impact on soil pH. However, the application of biochar at doses of 2%, 4%, and 8% significantly increased soil pH, and a result of this treatment was more visible in acidic soils with a pH ≤ 6.2 (pH increase to 1.17 units) than in neutral soils with a pH > 6.2 (pH increase to 0.4 units). Various effects of biochar on soil pH could result from the properties of this material, regulated by raw material parameters, as well as the temperature and other conditions of the pyrolysis—the process in which it was created [48]. Biochar’s ability to increase soil pH could result mainly from the presence of alkaline compounds in this material, including ash and carbonates, its surface properties, and the ability to reduce exchangeable acid ions (Al^3+^ and H^+^) [47].

### 3.2. Sulfate Sulfur Content

Elemental sulfur is the most concentrated source of sulfur for plants. However, to become plant available, it must be converted to sulfate ion S-SO_4_^2−^. Therefore, it is important to know the factors shaping this process, and an incubation study could be a valuable tool for this purpose. Ultimately, to acquire a complete knowledge about the effectiveness of fertilizers used, these materials should also be assessed under field conditions [49,50].

On the day of the sulfur pulp and organic materials application, sulfate sulfur content in very light soil ranged from 2.25 to 5.47 mg S/kg d.m., and in heavy soil, it ranged from 6.36 to 8.94 mg S/kg d.m. (Table 4). During incubation, the sulfate sulfur content in both soils increased (this concerned all experimental treatments with the addition of tested materials). After 240 days of experiment, sulfate sulfur content in the very light and heavy soil amounted from 2.40 to 23.51 mg S/kg d.m. and from 6.79–29.46 mg S/kg d.m., respectively (Table 4).

The sulfate sulfur content in the very light and heavy soil with the addition of sulfur pulp and its mixtures with organic materials was increased significantly until the 60th day of incubation. The content of this element determined at later sampling dates (120th and 240th day) was, as a rule, significantly lower than the content of sulfate sulfur determined on day 60. Throughout the incubation period, the treatment with the addition of a mixture of sulfur pulp at the SII sulfur dose and digestate (in both very light and heavy soil) was characterized by the highest sulfate sulfur content. During the incubation period, no statistically significant change in this parameter value of very light soil control treatment (without additives) was found. In the heavy soil control treatment, changes in the sulfate sulfur content were slight.

After 240 days of the experiment, the treatments of very light and heavy soil with the addition of sulfur pulp and its mixtures with organic materials had a significantly higher content of sulfate sulfur than the control treatment (without additives). Among the treatments with the tested materials in very light soil, the treatment with the addition of sulfur pulp at the SII sulfur dose and digestate significantly had the highest sulfate ions content, while significantly the lowest—treatments with sulfur pulp at the SI and SII sulfur doses and with mixtures of sulfur pulp at the SI sulfur dose with organic materials. Concerning the content of sulfate ions in heavy soil treatments after 240 days of incubation, the treatments with the addition of sulfur pulp at the SII sulfur dose and its mixtures with manure and digestate had significantly the highest sulfate ions content, while significantly the lowest—the treatments with the addition of sulfur pulp at the SI sulfur dose and its mixtures with organic materials.

After conducting the experiment, the effect of the sulfur dose on the sulfate sulfur content of both tested soils (regardless of organic material addition) was determined. Throughout the incubation period, sulfate ions content in the treatments with the addition of sulfur pulp at the SII sulfur dose and its mixtures with organic materials was comparable to or significantly higher than the sulfate ions content in the treatments with the addition of sulfur pulp at the SI sulfur dose and its mixtures with organic materials.

In relation to the fertilizing treatments with only the sulfur pulp, a beneficial effect of organic materials on the sulfate sulfur content of both tested soils was found. A significantly higher value of this parameter was found, especially in the treatments with the double sulfur dose (SII).

The findings presented in this study clearly indicate that the application of sulfur pulp and its mixtures with organic materials affected the sulfate sulfur content of both tested soils. After 240 days from the application of tested materials, in relation to the control treatment, the sulfate sulfur content in the very light and heavy soil increased. Similar results were presented by Wen et al. [51], Yang et al. [33], Degryse et al. [50] and Tabak et al. [35]. The cited authors also highlighted that the transformation of elemental sulfur to sulfate ions constitutes a gradual process. Efficiency and the rate of elemental sulfur conversion to sulfate ions are shaped by the size of introduced elemental sulfur particles and their dispersion in soil particles, as well as soil properties (temperature, humidity, pH value, structure, and the abundance of organic matter), which then affects the number, structure, and activity of the elemental sulfur-oxidizing microorganisms’ population [51,52,53]. Similar to the presented findings, decreasing sulfate sulfur content after reaching a maximal level (on the 60th day of incubation) was also noted by Jaggi et al. [53]. As the authors highlighted, this could be related to the changing activity of soil microorganisms. Higher sulfate sulfur content after the introduction of elemental sulfur in combination with organic materials, in comparison to the application of elemental sulfur alone, was also reported by Cifuentes and Lindemann [54]. As it was stated in this paper, soil amendment with elemental sulfur significantly increased its abundance in a readily available sulfate form. Although sulfurs are an essential nutrient, their excess in the environment could pose harm to plants and cause soil and water pollution [51,55]. Double sulfur doses used in the presented study (40 mg S/kg d.m. of soil and 60 mg S/kg d.m. of soil) were high, changing the level of sulfate sulfur content in soil from low to elevated as a result of human pressure (according to the Polish guidelines). Therefore, taking into account the practical aspects, it is not recommended to introduce sulfur pulp in such high doses, as it could pose a threat to the environment [56,57].

### 3.3. Total Organic Carbon Content

Soil organic matter constitutes a heterogeneous mixture composed of living organisms inhabiting soil, and organic remains (plant and animal) at various stages of decomposition. This component shapes many functions of the soil environment. Resources of organic matter are considered a quality indicator of the soil environment. Any change in the amount of this resource indicates the correctness of agricultural production management (or its absence).

After 240 days of incubation, the total organic carbon content in treatments of very light and heavy soil with the addition of tested materials was, respectively, comparable and comparable or significantly higher than the value of this parameter determined in the control treatment (without additions). In the control treatment, the total organic carbon content amounted to 4.70 g C/kg d.m. and 10.91 g C/kg d.m. for very light and heavy soil, respectively (Table 5). In both soils, among treatments with the addition of sulfur pulp and its mixtures with organic materials, statistical differences in these component values were small and related only to some treatments. In heavy soil, a significant change in the total organic carbon content (increase) was found after the application of mixtures of sulfur pulp at the SI and SII sulfur doses with biochar (11.80 g C/kg d.m. and 11.83 g C/kg d.m.), as well as a mixture of sulfur pulp at the SI sulfur dose with manure (11.87 g C/kg d.m.), compared to the control treatment (Table 5).

Following the experiment, it was stated that the impact of the sulfur dose on the total organic carbon content of both soils was small (regardless of organic material addition). Throughout the incubation period, the total organic carbon content in treatments with the addition of sulfur pulp at dose SII and its mixtures with organic materials were comparable to the values of this parameter determined in treatments with the addition of sulfur pulp at dose SI and its mixtures with organic materials.

As a rule, no statistically significant relationship between the total organic carbon content in the very light soil and the presence of organic materials was recorded (compared to the treatments amended only with sulfur pulp). By contrast, in the heavy soil, a significant increase in the content of this component was found after the introduction of organic materials (this concerned the treatments with the addition of mixtures of sulfur pulp at the SI sulfur dose with organic materials).

The findings presented in this study, therefore, indicate that the impact of the applied sulfur pulp and its mixtures with organic materials on the total organic carbon content of both incubated soils was slight. A minor effect of the applied materials on organic carbon content in incubated soil was also presented by Tambone and Adani [41]. The authors stated that, throughout 90 days of incubation, treatments with the addition of organic materials (compost from lignocellulosic residues and the organic fraction of municipal solid waste, digestate, and stabilized sewage sludge) had a higher content of organic carbon than the control treatment (with no additions) and the treatment with the addition of urea (organic carbon content in these treatments amounted from 13.6 to 15.3 g C/kg d.m.). However, the determined differences were statistically insignificant and related only to some treatments. The authors also highlighted that the organic carbon content was smaller the longer the experiment lasted (regardless of the fluctuations in some sampling dates). Opposite results were presented by Olowoboko et al. [58]. The authors, after conducting an 8-week incubation experiment, found that the addition of dried manure (cattle, goat, and chicken), ash from these materials, and mineral fertilization with NPK, as a rule, led to an increase in organic carbon content, in comparison to the control treatment (without additives). The change in the total organic carbon content is a sensitive indicator of the transformations occurring in soil, describing the intensity of the processes taking place, and indicating the shifts in organic matter resources.

### 3.4. Dehydrogenase Activity

The functionality of such a complex and dynamic ecosystem as the soil is shaped by interactions between chemical, physical, and biological properties [59]. Microorganisms are an inseparable element of this ecosystem, shaping its quality and productivity [60]. An important indicator of soil fertility is dehydrogenase activity. It is determined by the metabolic rate of soil microorganisms. These metabolic processes are linked to the mineralization of organic matter, resulting in the release of significant amounts of nutrients into the soil solution. The level of dehydrogenase activity also indicates whether the physicochemical, chemical, and physical properties of the soil are suitable for living organisms.

Croplands are characterized by high spatial variability, which could affect the activity and species composition of fauna and flora occurring in this habitat. Microbial diversity is an essential factor to maintain and improve soil quality, which is a complex ecosystem inhabited by a large number of microorganisms [61]. Assessment of microorganisms activity is a useful tool to determine soil condition. Natural factors and used agricultural practices contribute to every component of its fertility, especially microbiological activity.

On the day of sulfur pulp and organic materials application, DHA activity in the very light soil ranged from 0.82 to 1.65 μg TPF/g d.m./24 h, and in heavy soil, it ranged from 13.00 to 19.62 μg TPF/g d.m./24 h (Table 6). During incubation, the DHA activity in both soils decreased (this concerned all experimental treatments (Table 6). After 120 days of the experiment, DHA activity in the very light soil ranged from 0.39–0.84 μg TPF/g d.m./24 h, and in the heavy soil, it ranged from 2.33 to 9.49 μg TPF/g d.m./24 h (Table 6). Due to decreasing DHA activity and its low value determined on the 120th day of incubation, the collection of soil samples for laboratory analyses on the last planned sampling date (day 240) was abandoned.

The activity of DHA in tested soils, after the application of sulfur pulp and its mixtures with organic materials, decreased significantly on the 60th and 120th day of incubation. The determined value of the discussed parameter was, as a rule, significantly lower than the DHA activity determined in the previous sampling dates (0, 15, 30). It should be noted, however, that a significant decrease in DHA activity, in some very light soil experimental treatments, was found already on the 15th day of incubation. In the heavy soil, DHA activity fluctuations were observed until the 30th day of incubation (DHA activity in some treatments decreased or increased significantly; a visible increase was found, especially in the treatment with the addition of a mixture of sulfur pulp at the SI sulfur dose with digestate). Throughout the experimental period, DHA activity in the very light soil control treatment (with no additions) was low, and no statistically significant changes in this parameter were recorded. In the heavy soil control treatment (with no additions), a statistically significant change (decrease) in DHA activity was found on the 60th and 120th days of incubation.

After 120 days of incubation, treatments of very light and heavy soil with the addition of sulfur pulp and its mixtures with organic materials resulted in comparable or significantly lower DHA activity than the control treatments (without additives). After conducting the experiment, statistically significant differences in DHA activity of both soils, resulting from the application of the tested materials, were relatively small and related only to some objects. In the very light soil, in relation to the control treatment, a significant change in the activity of the discussed enzyme (reduction) was found after the application of the mixtures of sulfur pulp at the SII sulfur dose with digestate and biochar. In the heavy soil, in relation to the control treatment, a significant change in DHA activity (reduction) was found in all experimental treatments. The treatment with the addition of the mixtures of sulfur pulp at the SI sulfur dose with digestate and biochar was an exception to this rule.

After conducting the experiment, a minor effect of the sulfur dose on DHA activity of both tested soils (regardless of organic material addition) was observed. Throughout the incubation period, DHA activity in treatments with the addition of sulfur pulp at the SII sulfur dose and its mixtures with organic materials was comparable to or significantly lower than the values of this parameter determined in the treatments with the addition of sulfur pulp at the SI sulfur dose and its mixtures with organic materials.

In relation to the treatment of fertilizers only with sulfur pulp, a beneficial effect of organic materials (manure and digestate) on DHA activity in the initial incubation period (days 0–30) was found. The value of this parameter at later sampling dates (60th and 120th day) was, as a rule, not affected by the addition of organic materials.

The presented findings indicate that the application of sulfur pulp and its mixtures with organic materials affected the dehydrogenase activity of the tested soils. The intensity of this effect depended on the soil granulometric composition and the length of the incubation period. In the initial period of incubation (days 0–30), a beneficial effect of mixtures of sulfur pulp at the SI and SII sulfur doses with manure and digestate was found. The addition of these materials enhanced DHA activity in both very light and heavy soils. Treatments with the mentioned additions were characterized, as a rule, by a significantly higher DHA activity than the control treatments. As the incubation time went on, a reduction in dehydrogenase activity was stated. Similar observations were presented also by Mierzwa-Hersztek et al. [62] and Tabak et al. [35].

Various effects of applied materials on soil enzymatic activity have been presented by other authors. Tabak et al. [35] showed a positive influence of sulfur pulp on DHA activity and highlighted that the intensity of this effect depended on the soil pH and incubation experiment duration. The authors also found a sharp decrease in this parameter during the first 15 days of incubation, which progressed over time. Gupta et al. [63], after five years of annual application of elemental sulfur at two doses (22 kg/ha/year or 44 kg/ha/year), found a decrease in DHA activity (at given doses, values of DHA activity amounted to 51.4 µg TPF/g/24 h and 19.7 µg TPF/g/24 h, respectively, while DHA activity of the control treatment was 95.3 µg TPF/g/24 h). By contrast, Yang et al. [39] showed that repeated application of S0 increases the abundance and activity of the soil organisms population. Siwik-Ziomek and Szczepanek [64] highlighted that DHA activity presents sensitivity to NPKS mineral fertilization. This treatment increased biomass production, and indirectly, also the amount of plant root secretions, and thus enhanced the biochemical soil activity. Furthermore, the authors concluded that the increase in the nitrogen dose (from 144 kg N/ha to 180 kg N/ha) resulted in a decrease in soil microorganisms’ activity. This can be explained by the accumulation of toxic ammonia or a reduction in the soil pH [64,65]. What is more, a decrease in soil DHA activity could result from a shortage of easily degradable carbon substrates and a depletion of nutrient resources [66,67,68]. Ros et al. [69] reported that, after the depletion of organic matter resources, soil microbes’ activity is primarily maintained by plant root secretions. In such a situation, the mentioned substances constitute a key source of energy. The authors presented that after soil fertilization with organic materials (fresh biomass of municipal organic waste, municipal organic waste compost, and a mixture of fresh biomass of municipal organic waste with straw), DHA activity, during two years of a field experiment, increased significantly, in relation to the control treatment (without fertilization). Moreover, to maintain a high level of soil enzymatic activity, apart from a high content of organic matter, its quality is equally important, as it constitutes a source of energy for microorganisms during their development and enzyme production. Moreover, Garcia-Gil et al. [70] found that soil DHA activity was shaped by applied fertilization, and its value was arranged in the following order: manure < municipal solid waste compost < control treatment (without fertilization) < mineral NPK fertilization. Shah et al. [71], after conducting an incubation experiment, found that soil microbiological activity increased with increasing doses of biochar (0, 5, 10, and 20 Mg/ha).

## 4. Conclusions

As demonstrated in the study, waste sulfur has a high fertilizing potential. The material can be used to enrich soils with sulfur, to decrease the environmental and economic burden caused by waste disposal. The method of biogas purification involving the use of iron and EDTA ligand is often used in sewage treatment plants. The method, however popular, is not followed by the environmental application of waste, despite its high content of elemental sulfur. Recycling sulfur corresponds with the growing trend of zero-waste technologies in all branches of the economy. Based on our knowledge, there is a scarcity of reports focused on the use of waste sulfur and its mixtures with organic materials for fertilization and fertilizer production. Thus, the presented findings constitute a valuable tool to assess the agricultural potential of discussed waste material and identify a way for its reuse.

Using the waste sulfur significantly increased the soil sulfate sulfur content. This indicated its high potential as a raw material for fertilizer production. A factor to keep in mind when using waste sulfur is its significant potential for soil acidification. The applied material did not have an effect on the level of dehydrogenase activity.

## Figures and Tables

**Table 1:** Selected properties of soil material before establishing the experiment.

Parameter	Very Light Soil	Heavy Soil
Soil texture, %		
1.0–0.1	85	10
0.1–0.02	12	70
<0.02	3	20
Maximum water capacity, %	20.5	43.9
pH_H2O_	6.01	5.42
pH_KCl_	5.04	4.35
Hydrolityc acidity, mmol (+)/kg d.m.	14.9	41.6
Sulfate S, mg/kg d.m.	3.57	10.37
Total N, g/kg d.m.	0.605	1.59
Total C, g/kg d.m.	8.23	19.7
Total S, mg/kg d.m.	97.6	231
Total Fe, mg/kg d.m.	2.70 × 10^3^	7.56
Total Cd, mg/kg d.m.	0.683	0.792
Total Cr, mg/kg d.m.	3.86	16.8
Total Cu, mg/kg d.m.	4.27	9.48
Total Hg, mg/kg d.m.	traces	traces
Total Mn, mg/kg d.m.	106	396
Total Ni, mg/kg d.m.	3.18	6.74
Total Pb, mg/kg d.m.	7.13	25.5
Total Zn, mg/kg d.m.	23.9	67.6

**Table 2:** Selected chemical properties of materials used in the incubation experiment.

Parameter	Sulfur Pulp	Manure	Digestate	Biochar
d.m., %	32.3	93.7	25.1	98.7
Organic matter	-	652	552	954
Total N, g/kg d.m.	4.19	31.3	35.7	4.63
Total C, g/kg d.m.	20.3	330	311	516
Total Na, g/kg d.m.	12.2	1.98	0.700	0.125
Total Mg, g/kg d.m.	traces	5.33	3.89	1.06
Total K, g/kg d.m.	traces	10.4	2.37	2.40
Total Ca, g/kg d.m.	traces	46.2	26.9	5.34
Total P, g/kg d.m.	traces	13.9	19.0	0.834
Total Fe, g/kg d.m.	2.95	1.68	9.09	0.999
Total S, g/kg d.m.	864	5.19	9.00	0.199
Total Cd, mg/kg d.m.	traces	0.225	1.46	0.025
Total Cr, mg/kg d.m.	traces	7.34	51.5	46.9
Total Cu, mg/kg d.m.	traces	57.0	169	6.83
Total Mn, mg/kg d.m.	traces	377	337	363
Total Ni, mg/kg d.m.	traces	4.42	24.6	26.4
Total Pb, mg/kg d.m.	traces	1.05	22.2	0.816
Total Hg, mg/kg d.m.	not determined	traces	0.659	traces
Total Zn, mg/kg d.m.	traces	266	716	42.9

**Table 3:** Very light and heavy soil pH_KCl_ value throughout the incubation experiment.

Treatment *	Number of Incubation Days					
	0	15	30	60	120	240
	Very Light Soil					
C	5.78 ^stuvwx^ **	5.80 ^tuvwxy^	5.77 ^stuvwx^	5.70 ^qrstuvw^	5.55 ^jklmnop^	5.41 ^efgh^
SI	5.76 ^rstuvwx^	5.64 ^opqrst^	5.56 ^klmnopq^	5.52 ^ijklmno^	5.44 ^efghi^	5.13 ^b^
SII	5.76 ^rstuvwx^	5.47 ^fghijkl^	5.45 ^fghij^	5.45 ^fghij^	5.36 ^de^	5.10 ^b^
SI + M	6.02 ^xy^	5.80 ^tuvwxy^	5.69 ^pqrstuv^	5.57 ^lmnopq^	5.53 ^ijklmno^	5.26 ^c^
SI + D	5.91 ^vwxy^	5.61 ^nopqrs^	5.56 ^lmnopq^	5.48 ^fghijklm^	5.45 ^fghijk^	5.11 ^b^
SI + B	5.85 ^uvwxy^	5.77 ^stuvwx^	5.68 ^pqrstu^	5.52 ^ijklmno^	5.51 ^ijklmno^	5.31 ^cd^
SII + M	6.09 ^y^	5.79 ^tuvwx^	5.69 ^pqrstuvw^	5.60 ^mnopqr^	5.49 ^ghijklmn^	5.28 ^c^
SII + D	5.92 ^wxy^	5.56 ^klmnopq^	5.44 ^efghi^	5.39 ^ef^	5.27 ^c^	5.02 ^a^
SII + B	5.81 ^tuvwxy^	5.55 ^jklmnopq^	5.50 ^hijklmn^	5.40 ^efg^	5.31 ^cd^	5.05 ^a^
	**Heavy Soil**					
C	4.95 ^nopqrs^ **	5.00 ^pqrst^	4.88 ^klmnop^	4.82 ^jklm^	4.73 ^fghi^	4.50 ^b^
SI	5.02 ^qrst^	4.94 ^nopqrs^	4.85 ^klmn^	4.74 ^ghij^	4.70 ^efg^	4.45 ^a^
SII	4.95 ^nopqrs^	4.96 ^nopqrs^	4.81 ^ijklm^	4.70 ^efg^	4.63 ^d^	4.42 ^a^
SI + M	5.12 ^t^	5.07 ^rst^	4.94 ^nopqrs^	4.89 ^lmnop^	4.80 ^ijkl^	4.56 ^c^
SI + D	5.08 ^rst^	4.97 ^opqrst^	4.85 ^klmn^	4.79 ^hijk^	4.71 ^efg^	4.51 ^b^
SI + B	5.04 ^qrst^	4.93 ^nopqr^	4.87 ^klmno^	4.75 ^ghij^	4.71 ^efgh^	4.43 ^a^
SII + M	5.08 ^st^	4.99 ^opqrst^	4.85 ^klmn^	4.80 ^ijkl^	4.73 ^ghi^	4.50 ^b^
SII + D	5.03 ^qrst^	4.90 ^mnopq^	4.85 ^klmn^	4.72 ^fgh^	4.65 ^de^	4.44 ^a^
SII + B	4.98 ^opqrst^	4.94 ^nopqrs^	4.82 ^jklm^	4.71 ^efgh^	4.66 ^def^	4.43 ^a^

* according to experimental design (see Section 2.2. of this paper), ** mean values marked with the same letters do not differ statistically significantly at the significance level of *p* ≤ 0.05, according to Duncan’s test.

**Table 4:** Sulfate sulfur content in very light and heavy soil throughout the incubation experiment (mg S/kg d.m. ± SD).

Treatment *	Number of Incubation Days					
	0	15	30	60	120	240
	Very Light Soil					
C	2.25 ^a^ ** ± 0.41	1.58 ^a^ ± 0.23	2.18 ^a^ ± 0.19	4.28 ^abc^ ± 0.93	2.81 ^abc^ ± 0.11	2.40 ^ab^ ± 0.36
SI	2.29 ^ab^ ± 0.36	5.82 ^abcde^ ± 0.70	11.76 ^ghij^ ± 3.36	14.88 ^hijklmnop^ ± 1.96	12.10 ^ghijk^ ± 1.33	9.44 ^efg^ ± 0.49
SII	2.82 ^abc^ ± 0.56	13.98 ^hijklmn^ ± 1.4	18.29 ^nopq^ ± 0.99	19.36 ^pq^ ± 6.11	21.31 ^qr^ ± 3.40	13.55 ^ghijklm^ ± 0.21
SI + M	4.32 ^abc^ ± 1.06	6.83 ^bcdef^ ± 0.05	15.80 ^ijklmno^ ± 1.95	18.15 ^nopq^ ± 1.74	14.63 ^hijklmno^ ± 1.75	12.04 ^ghijk^ ± 2.09
SI + D	5.47 ^abcde^ ± 0.17	9.19 ^defg^ ± 1.19	11.25 ^ghi^ ± 0.30	15.45 ^ijklmno^ ± 1.97	15.25 ^ijklmno^ ± 2.64	13.40 ^ghijklm^ ± 0.96
SI + B	3.09 ^abc^ ± 0.22	7.03 ^cdef^ ± 2.30	12.40 ^ghijkl^ ± 0.67	16.14 ^jklmno^ ± 3.33	16.40 ^klmnop^ ± 1.91	10.62 ^fgh^ ± 0.98
SII + M	5.02 ^abcd^ ± 0.10	15.93 ^jklmno^ ± 3.40	25.20 ^rs^ ± 4.44	39.04 ^t^ ± 3.52	17.00 ^lmnopq^ ± 1.15	18.62 ^opq^ ± 1.84
SII + D	4.86 ^abcd^ ± 0.77	25.03 ^rs^ ± 3.33	27.37 ^s^ ± 4.34	35.48 ^t^ ± 6.50	23.80 ^rs^ ± 2.31	23.51 ^rs^ ± 3.79
SII + B	3.65 ^abc^ ± 0.41	15.36 ^ijklmno^ ± 2.85	13.21 ^ghijklm^ ± 2.21	17.38 ^mnopq^ ± 3.86	17.40 ^mnopq^ ± 1.83	19.41 ^pq^ ± 0.42
	**Heavy Soil**					
C	6.36 ^ab^ ** ± 0.08	6.64 ^ab^ ± 0.94	8.97 ^bcd^ ± 0.47	9.69 ^cde^ ± 0.30	5.26 ^a^ ± 0.78	6.79 ^ab^ ± 0.88
SI	6.44 ^ab^ ± 0.09	9.83 ^cdef^ ± 0.97	20.73 ^mn^ ± 0.88	29.64 ^rstu^ ± 2.99	16.42 ^jk^ ± 1.05	15.54 ^ij^ ± 1.84
SII	8.46 ^bc^ ± 0.43	13.46 ^ghi^ ± 1.03	39.32 ^vw^ ± 1.16	50.61 ^y^ ± 1.95	29.09 ^rst^ ± 0.42	25.81 ^pq^ ± 0.95
SI + M	8.67 ^bc^ ± 0.66	12.13 ^efg^ ± 0.60	28.9 ^rs^ ± 2.28	38.03 ^v^ ± 2.43	19.98 ^lmn^ ± 1.99	15.19 ^hij^ ± 0.84
SI + D	8.94 ^bcd^ ± 0.49	11.59 ^defg^ ± 0.82	24.18 ^op^ ± 1.43	28.21 ^qrs^ ± 0.56	17.68 ^jkl^ ± 0.61	15.16 ^hij^ ± 0.31
SI + B	6.86 ^abc^ ± 0.16	8.96 ^bcd^ ± 0.36	23.56 ^op^ ± 2.20	28.31 ^qrs^ ± 2.12	17.10 ^jk^ ± 2.28	16.78 ^jk^ ± 0.69
SII + M	8.43 ^bc^ ± 0.32	12.64 ^gh^ ± 0.61	41.16 ^w^ ± 2.81	50.83 ^y^ ± 3.15	30.14 ^stu^ ± 2.25	26.99 ^qr^ ± 1.11
SII + D	7.16 ^abc^ ± 0.33	18.73 ^klm^ ± 0.99	43.89 ^x^ ± 2.56	53.55 ^z^ ± 2.04	32.36 ^u^ ± 0.80	29.46 ^rst^ ± 1.02
SII + B	7.57 ^abc^ ± 0.38	12.54 ^fgh^ ± 0.72	38.07 ± 3.78	49.65 ^y^ ± 3.07	31.88 ^tu^ ± 2.17	21.96 ^no^ ± 1.82

* according to experimental design (see Section 2.2. of this paper), ** mean values marked with the same letters do not differ statistically significantly at the significance level of *p* ≤ 0.05, according to Duncan’s test.

**Table 5:** Total organic carbon content in very light and heavy soil after 240 days of incubation experiment (g C/kg d.m. ± SD).

Treatment *	Very Light Soil	Heavy Soil
C	4.70 ^abc^ ** ± 0.35	10.91 ^ab^ ± 0.48
SI	4.42 ^ab^ ± 0.29	10.60 ^a^ ± 0.25
SII	4.37 ^ab^ ± 0.37	11.22 ^abc^ ± 0.35
SI + M	4.62 ^abc^ ± 0.32	11.87 ^c^ ± 0.65
SI + D	4.24 ^a^ ± 0.16	11.61 ^bc^ ± 0.65
SI + B	4.27 ^a^ ± 0.23	11.80 ^c^ ± 0.22
SII + M	5.11 ^c^ ± 0.35	11.44 ^bc^ ± 0.44
SII + D	4.91 ^bc^ ± 0.38	11.61 ^bc^ ± 0.29
SII + B	4.66 ^abc^ ± 0.30	11.83 ^c^ ± 0.12

* according to experimental design (see Section 2.2. of this paper), ** mean values marked with the same letters do not differ statistically significantly at the significance level of *p* ≤ 0.05, according to Duncan’s test.

**Table 6:** Dehydrogenase activity in very light and heavy soil throughout the incubation experiment (μg TPF/g d.m./24h ± SD).

Treatment *	Number of Incubation Days				
	0	15	30	60	120
	Very Light Soil				
C	0.95 ^ghijk^ ** ± 0.07	0.96 ^ghijk^ ± 0.13	0.92 ^ghij^ ± 0.03	0.78 ^defgh^ ± 0.06	0.72 ^cdefg^ ± 0.05
SI	0.82 ^defgh^ ± 0.10	0.82 ^defgh^ ± 0.01	0.92 ^ghij^ ± 0.05	0.93 ^ghij^ ± 0.14	0.67 ^bcdef^ ± 0.01
SII	0.86 ^fghi^ ± 0.04	0.91 ^ghij^ ± 0.02	0.74 ^cdefg^ ± 0.12	0.65 ^bcdef^ ± 0.04	0.52 ^abc^ ± 0.02
SI + M	1.33 ^mn^ ± 0.24	1.12 ^jklm^ ± 0.12	1.01 ^hijkl^ ± 0.16	0.94 ^ghij^ ± 0.09	0.84 ^efghi^ ± 0.05
SI + D	1.65 ^o^ ± 0.39	1.42 ^n^ ± 0.15	1.17 ^klm^ ± 0.28	0.85 ^efghi^ ± 0.07	0.67 ^bcdef^ ± 0.06
SI + B	0.82 ^defgh^ ± 0.04	0.83 ^defghi^ ± 0.07	0.73 ^cdefg^ ± 0.09	0.62 ^bcde^ ± 0.04	0.51 ^abc^ ± 0.04
SII + M	1.52 ^no^ ± 0.23	1.21 ^lm^ ± 0.14	1.21 ^lm^ ± 0.15	0.65 ^bcdef^ ± 0.01	0.60 ^bcd^ ± 0.04
SII + D	1.10 ^jkl^ ± 0.13	1.06 ^ijkl^ ± 0.15	1.01 ^hijkl^ ± 0.07	0.62 ^bcde^ ± 0.09	0.45 ^ab^ ± 0.06
SII + B	0.82 ^defgh^ ± 0.06	0.83 ^defghi^ ± 0.04	0.66 ^bcdef^ ± 0.05	0.63 ^bcdef^ ± 0.02	0.39 ^a^ ± 0.02
	**Heavy Soil**				
C	14.70 ^mnopq**^ ± 0.85	15.70 ^nopqr^ ± 2.15	17.00 ^pqrst^ ± 0.20	10.01 ^fghi^ ± 1.38	9.49 ^efgh^ ± 2.47
SI	13.92 ^klmnop^ ± 1.50	16.50 ^pqrs^ ± 2.48	13.19 ^jklmno^ ± 1.06	13.00 ^ijklmno^ ± 1.91	5.53 ^bcd^ ± 0.41
SII	14.73 ^mnopq^ ± 2.29	15.30 ^nopq^ ± 2.31	10.47 ^ghij^ ± 1.07	8.22 ^defg^ ± 1.10	5.27 ^bcd^ ± 0.64
SI + M	14.46 ^lmnopq^ ± 0.30	19.59 ^tu^ ± 0.20	20.81 ^u^ ± 2.86	11.80 ^hijklm^ ± 0.74	5.00 ^abc^ ± 0.48
SI + D	16.10 ^opqr^ ± 2.27	21.10 ^u^ ± 1.20	31.97 ^v^ ± 4.56	9.50 ^efgh^ ± 0.47	8.14 ^defg^ ± 0.49
SI + B	15.92 ^nopqr^ ± 0.54	17.09 ^qrst^ ± 0.82	11.05 ^ghijk^ ± 1.02	11.83 ^hijklm^ ± 0.32	6.67 ^cde^ ± 0.42
SII + M	17.16 ^qrst^ ± 0.87	21.19 ^u^ ± 2.49	18.37 ^rstu^ ± 2.93	13.21 ^jklmno^ ± 2.02	5.92 ^bcd^ ± 0.89
SII + D	19.62 ^tu^ ± 1.50	19.30 ^stu^ ± 1.89	15.07 ^nopq^ ± 0.71	9.12 ^efgh^ ± 2.15	3.43 ^ab^ ± 0.08
SII + B	13.00 ^ijklmno^ ± 1.99	12.79 ^ijklmn^ ± 0.03	11.50 ^hijkl^ ± 0.98	7.07 ^cdef^ ± 0.43	2.33 ^a^ ± 0.17

* according to experimental design (see Section 2.2. of this paper), ** mean values marked with the same letters do not differ statistically significantly at the significance level of *p* ≤ 0.05, according to Duncan’s test.

## Data Availability

Not applicable.
